# Forty-Third Annual Report on the State of the Asylum for the Relief of Persons Deprived of the Use of Their Reason

**Published:** 1860-07

**Authors:** 


FORTY-THIRD ANNUAL REPORT on the state of the Asylum for the relief of persons deprived of the use of their reason. Philadelphia: 1860.

This is a pamphlet of 31 pages, containing the statistics and rules of the Friends Asylum for the Insane, in Philadelphia. A good report from an excellent institution.

